# Ethnic Variation in Breastfeeding and Complimentary Feeding in the Republic of Ireland

**DOI:** 10.3390/nu6051832

**Published:** 2014-05-02

**Authors:** Patricia Dominguez Castro, Richard Layte, John Kearney

**Affiliations:** 1 Trinity College Dublin, College Green, Dublin 2, Ireland; 2 Economic and Social Research Institute, Sir John Rogerson’s Quay, Dublin 2, Ireland; E-Mail: Richard.Layte@esri.ie; 3 Dublin Institute of Technology, Kevin Street, Dublin 8, Ireland; E-Mail: John.Kearney@dit.ie

**Keywords:** infant feeding, breastfeeding, complimentary feeding, acculturation

## Abstract

Early nutrition plays a pivotal role in long-term health. The World Health Organization (WHO) recommends exclusive breastfeeding during the first six months of life, with the gradual introduction of solids after this period. However, studies in the Republic of Ireland (ROI) have shown poor compliance with guidelines. The ROI continues to have one of the lowest breastfeeding rates worldwide. Our objective was to analyse differences in breastfeeding and complimentary feeding behaviours between Irish and non-Irish mothers residing in the ROI, as well as the role of acculturation on these behaviours, using the national longitudinal study, Growing Up in Ireland (GUI). Mothers (*n* = 11,134) residing in the ROI were interviewed when their infants were nine months of age. The percentage of Irish mothers who initiated breastfeeding was 49.5%, as opposed to 88.1% among the non-Irish cohort (*p* < 0.001). Breastfeeding initiation reduced from 89.4% of non-Irish mothers who had arrived within the last year to five years ago to 67.5% for those who had arrived 11 to >20 years ago (*p* < 0.001). Our results indicate that cultural differences are an important factor in shaping patterns of infant feeding in the ROI. Reviewing existing support and education policies for parents is required to achieve the implementation of desirable infant feeding practices.

## 1. Introduction

Early nutrition plays a pivotal role in long-term health. Breastfeeding has been shown to have a protective role in the development of being overweight, obesity and chronic diseases later in life [1,2,3]. The World Health Organization (WHO) recommends exclusive breastfeeding during the first six months of life of the infant, with the gradual introduction of complimentary foods after this period [4]. The European Society of Paediatric, Gastroenterology, Hepatology and Nutrition (ESPGHAN) recommends not to introduce complimentary foods before 17 weeks and no later than 26 weeks, while also giving the advice to commence the introduction of solids near six months of age [5]. Early complimentary feeding has been shown by studies to increase the risk of overweight and obesity during childhood and adulthood [6,7,8,9]. Moreover, the transition from milk to solid foods can have a life-long influence on dietary patterns [6,10,11,12]. The introduction of complimentary foods cannot be studied in isolation from the type of milk feeding early in life, as milk type influences the type of solid foods introduced and the timing of their introduction [13]. Studies on the predictors of early complimentary feeding have shown that breastfeeding reduces the likelihood of early solid food introduction [14,15,16,17,18].

In the Republic of Ireland (ROI), the Department of Health and Children updated their advice in 2003 to recommend adherence to the WHO advice of exclusive breastfeeding during the first six months of the infants’ life [19]. The new Infant Feeding Guidelines released by the Food safety Authority of Ireland (FSAI) in November, 2012, maintain the recommendation made by ESPGHAN [20]. Despite these guidelines, the ROI continues to have one of the lowest breastfeeding rates worldwide, and compliance with complimentary feeding guidelines is poor [21]. Irish studies show rates of exclusive breastfeeding for six months of less than 1%, with 75% of infants being introduced to complimentary feeding before 17 weeks, 22.6% of these being introduced prematurely by 12 weeks [15,22].

Rates of breastfeeding in Ireland have increased since 2004, but they are still below national targets, and a large percentage of this increase has been attributed to changes in maternal characteristics, such as older age and an increase in non-national mothers [23,24,25]. Previous studies in the ROI have pointed out different patterns of breastfeeding rates by maternal origin of birth [23,26,27]. However, we are not aware of any studies in the ROI analysing different patterns in complimentary feeding introduction by ethnic group. Given the fact that the time of complimentary feeding introduction seems to be linked to the type of milk feeding early in life and given the low breastfeeding rates in the ROI, it could be hypothesized that Irish born mothers are more likely to introduce complimentary foods early in the life of their infant, thus increasing the risk of their infants suffering adverse health effects in the short and long term. Moreover, acculturation of non-Irish mothers could play a role in their infant feeding practices. The aim of this paper is to study variation in breastfeeding rates and the timing of the introduction of complimentary feeding between Irish and non-Irish mothers living in the ROI, as well as the role of acculturation on these behaviours using cross-sectional data from the national longitudinal study of children in Ireland (Growing Up in Ireland).

## 2. Methods

### 2.1. Study Design and Sample

Growing Up in Ireland (GUI) is a nationally representative cohort study of nine-month-old infants residing in the Republic of Ireland. The main aim of the study is to study the factors affecting the lives of infants in Ireland with the aim of creating evidence-based policy. The study sample consisted of 11,134 nine-month-old infants who participated in the first wave of the GUI study. These were selected from the approximately 41,000 births over the period of 1 December 2007 to 30 June 2008. The completed sample of 11,134 represents approximately one-third of all births in the ROI over the field period. Families were invited to participate in the study when the child was nine months of age. The sampling frame for the project was the Child Benefit Register for the Republic of Ireland. Of 16,136 mothers selected from the sampling frame, 11,134 agreed to take part in the study, a response rate of 69% [28].

### 2.2. Questionnaires and Measurements

Primary caregivers, defined as the person who spent more time with the child, and secondary caregivers were interviewed at home and asked to complete a main questionnaire and a sensitive questionnaire. Since only 0.5% of the primary care givers nominated were not the biological mothers, we refer to responses from the primary care giver as those of the mother. Interviews were carried out using a mixture of CAPI (computer-assisted personal interviewing) and CASI (computer-assisted self-interviewing).

The wave one sample was selected from the Child Benefit Register for the Republic of Ireland, which was provided by the Department of Social Protection. Of 16,136 mothers selected from the sampling frame, 11,134 agreed to take part in the study, a response rate of 69%. Fieldwork was carried out over 7 months, extending from September 2008, to the end of April 2009. Children were selected so as to be 9-months-old at the time of the interview; consequently, eligible children were all those born between 1 December 2007 and 30 June 2008 [28].

The sampling frame for the study was the Child Benefit Register for the Republic of Ireland. The sample was selected on a systematic basis, pre-stratifying by marital status, county of residence, nationality and number of children (where child is defined as <16 years of age) in the household, using a random start and constant sampling fraction. The completed sample was statistically grossed or reweighted on the basis of external population estimates to ensure that it was wholly representative of all children aged one year or less in Ireland [28].

Interviewers measured and recorded both parents’ height and weight. A medically approved mechanical SECA 761 weighing scale was used for the adults’ weight and a Leicester measuring stick for their height. All stages of the Growing Up in Ireland project were subject to rigorous ethical review by a Research Ethics Committee convened by the Department of Children and Youth Affairs of the Irish Government. This included a review of all instrumentation, recruitment, consent and implementation protocols adopted at all stages of the study [28].

### 2.3. Statistical Analysis and Dependent Variable

The Statistical Package for the Social Sciences statistical software package version 19.0 (SPSS, Inc., Chicago, IL, USA) and STATA 13 (StataCorp LP, College Station, TX, USA) were used for the statistical analysis. Several independent variables considered as risk factors for early complimentary feeding were selected from the database. These included demographic factors, such as maternal age, maternal education, socioeconomic status and parity, and biological factors, such as maternal BMI, mode of delivery and infant’s health. In order to study the variations in complimentary feeding by ethnic group, as well as the influence of breastfeeding in its timing, the model was also adjusted for ethnicity, length of stay in the ROI, breastfeeding initiation and duration of exclusive breastfeeding. Data was analysed using cross-tabulations, and the χ^2^ statistical test, as well as multivariate binary logistic regression. Independent variables were included in the multivariate analysis if they were significant in the bivariate analysis.

Mothers were asked to report their ethnic or cultural background. The following options were provided; Irish, Irish traveller, any other white background, African, any other black background, Chinese, any other Asian background, and other, including mixed background. A recoded binary variable was constructed with two categories: Irish ethnic background and non-Irish ethnic background. This recoded variable was then combined with a variable that asked non-Irish mothers how long they had been residing in the ROI.

The dependent variable “early complimentary feeding” was constructed from a question in the database that asked mothers to indicate when they started to give their infants solid foods at least twice a day for several weeks. Solid foods were defined as baby cereals, pureed fruits, *etc*., and not milk or drinks. The dependent variable used is therefore the age at which complimentary feeding was established rather than the child’s age when solid foods were first introduced. Following ESPGHAN’s guidelines, a binary dependent variable was created with two categories <17 weeks for early complimentary feeding and ≥17 weeks for the acceptable introduction of complimentary feeding [5,28]. Statistical significance was taken as a *p*-value of < 0.05. The weights were on for all statistical analysis.

### 2.4. Definition of Covariates

Socio-economic status (SES) was assessed using three different indicators: household class, equivalised household income quintiles and household type. Income is equivalised to take into account household size and composition using the modified Organization for Economic Cooperation and Development equivalence scale (first adult value, 1; second or higher adults, 0.5; children aged < 14, 0.3). Primary and secondary caregivers were asked questions about their current occupation to derive the variable household class. Where the respondent was economically inactive (retired or unemployed) at the time of interview, previous employment was considered. The household class classification adopted was that used by the Central Statistics Office (CSO): professional workers, managerial and technical, non-manual, skilled-manual, semi-skilled, unskilled, all other gainfully occupied and unknown and never worked at all. This variable was recoded to contain only five categories: professional, managerial and technical workers; non-manual; skilled and semi-skilled manual; unskilled and all other gainfully occupied and unknown, and never worked at all. Household type is a fourfold variable derived from whether the study child is living in a one or two parent family, as well as the number of children (<18 years) living in the household. This resulted in a classification as follows: one parent, one child; one parent, two or more children; two parents, one child; two parents, two or more children [28].

Maternal education was coded as follows: no formal or primary education, secondary education and third-level education. Maternal age was coded as follows: ≤24, 25–34 and ≥35 years old. Measured parent BMI was classified according to the World Health Organization (WHO) classifications as underweight <18.5 kg/m^2^, normal weight ≥18.5 and <25 kg/m^2^, overweight ≥25 kg/m^2^ and <30 kg/m^2^ and obese ≥30 kg/m^2 ^ [28].

Mothers were asked about their infants’ overall health using the question: “In general, how would you describe the baby’s current health” with response categories “very healthy, no problems”, “healthy, but a few minor problems”, “sometimes quite ill” and “always unwell”. Children are defined as having been breastfed if they consumed breast milk at any stage regardless of the amount of time the baby was breastfed, including the colostrum in the first few days after birth. Exclusive breastfeeding was defined as the infant receiving only breast milk without any additional food or drink, regardless of the length of exclusive breastfeeding. The variable “duration of exclusive breastfeeding” was constructed from the question “how old was the baby when he/she stopped being exclusively breastfed” [28,29].

### 2.5. Missing Data

Some of the independent variables used in the analysis had a large percentage of missing cases: maternal BMI (5.1%) and equivalised household annual income (7.8%). This would have resulted in a proportion of the sample being lost from the analysis. In response, multiple imputation has been carried out in STATA 13 using the variables maternal age, maternal education, father’s education, household class, maternal employment status, current maternal smoking and migrant status to predict the missing values in maternal BMI and equivalised household annual income.

## 3. Results

### 3.1. Characteristics of the Study Cohort

Table 1 shows the characteristics of mothers disaggregated by ethnic group. The primary caregiver was defined as the person who spent the most time with the study infant. Irish mothers were younger on average than non-Irish (31.7 years with SD 5.3 compared to 30.9 with SD 5.4). Of those mothers with an Irish ethnic background, 49.5% initiated breastfeeding compared to 88.1% of those mothers with a non-Irish ethnic background. The mean duration of any breastfeeding for those that breastfed at all was 71.1 days (SD 66.4) for Irish mothers compared to 95.8 days (SD 69.2) for non-Irish. A higher percentage of mothers with an Irish ethnic background (15.5% *vs*. 7.6%) introduced complimentary foods early.

### 3.2. Percentage of Infants’ Breastfed and the Introduction of Complimentary Feeding in the <17 Weeks and ≥17 Weeks Categories Classified by Ethnic Group

**Figure 1:**
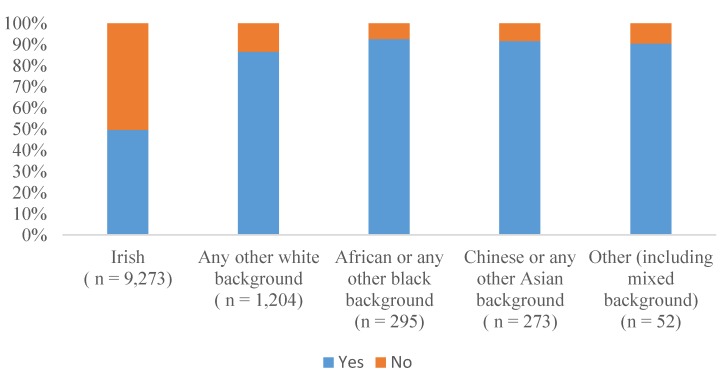
Breastfeeding initiation by ethnic group.

**Figure 2:**
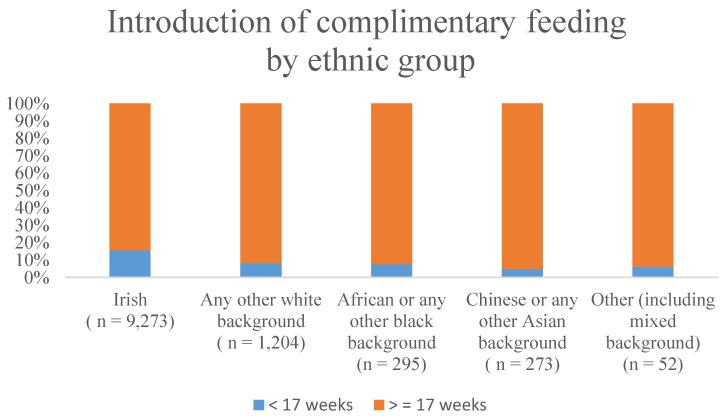
Introduction of complimentary feeding by ethnic group.

Figure 1 shows that the ethnic group with the highest percentage of mothers who breastfed their infants was African or any other black background, with 92.5% of mothers initiating breastfeeding. This group was followed by Chinese mothers, with 91.6% of breastfeeding initiation. Figure 2 shows the percentage of mothers introducing complimentary foods in the <17 weeks and ≥17 weeks categories classified by ethnic group. Fifteen-point-five percent of mothers with an Irish ethnic background introduced complimentary foods early (<17 weeks). The group with the lowest percentage of mothers introducing complimentary foods early (4.8%) was Chinese or any other Asian background.

**Table 1:** Characteristics of whole sample, national and non-national primary caregivers.

Characteristic	Primary Caregiver	Irish			Non-Irish		
		Sample (*n* *)	Mean or % ^†^	SD	Sample (*n* *)	Mean or % ^†^	SD
Mean age			31.7	5.3		30.9	5.4
BMI primary carer kg/m^2^	Underweight (less than 18.5)	9275	2.5		1859	3.7	
	Normal weight (18.5–24.9)		50.5			51.1	
	Overweight (25–29.9)		30.1			28.5	
	Obese (≥30)		16.8			16.7	
Having ever breastfed	Yes	9273	49.5		1859	88.1	
	No		50.5			11.9	
Having exclusively breastfed ^‡^	Yes	4592	39.3		1634	66.5	
	No		10.2			21.4	
Mean duration of any breastfeeding (days) ^‡^		4019	71.1	66.4	1153	95.8	69.2
Mean duration of exclusive breastfeeding (days) ^‡^		3556	74.7	62	1158	95.9	63.2
Introduction of complimentary feeding	<17 weeks	9151	15.5		1755	7.6	
	≥17 weeks		84.5			92.4	

* *n* provided is number of primary caregivers who answered each question; ^†^ Percentages provided are based on the total sample; ^‡^ mothers who reported not having ever breastfed were filtered out.

### 3.3. Predictors of Early Complimentary Feeding

Table 2 shows the adjusted model of significant factors that independently predicted early complimentary feeding introduction for the whole sample. After adjustment, the significant factors included the primary caregiver’s age, education, BMI, ethnicity and length of stay in the ROI, household class, household type and exclusive breastfeeding duration.

Non-Irish mothers who had been living in the ROI < 6 years were 50.7% less likely to introduce complimentary feeding early compared to Irish mothers (OR 0.493, 95% CI 0.371, 0.656). This protective effect of ethnicity decreased with the length of stay in the ROI, with non-Irish mothers who had been living in the ROI 11 to >20 years being 3.1% less likely to introduce complimentary feeding early when compared to Irish mothers (OR 0.969, 95% CI 0.613, 1.532).

Those mothers who exclusively breastfed >90 days were 93.9% less likely to introduce early complimentary feeding (OR 0.061, 95% CI 0.037, 0.101) when compared to those who did not exclusively breastfed.

### 3.4. Effects of Acculturation on Breastfeeding Initiation and Early Complimentary Feeding

**Figure 3:**
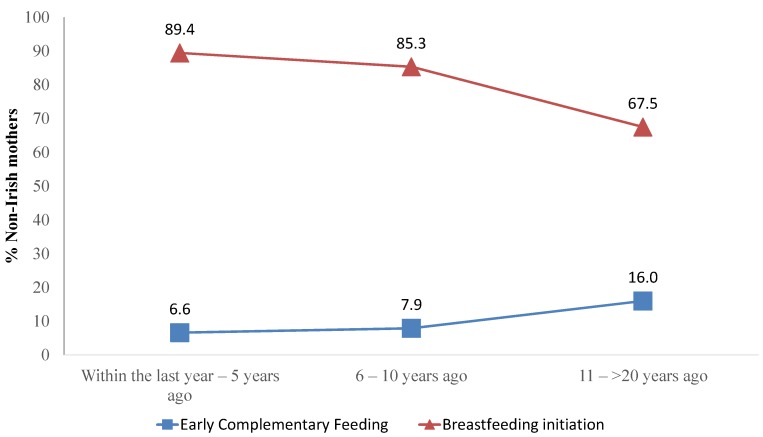
The effect of acculturation on breastfeeding initiation and early complimentary feeding.

Figure 3 shows the effects of acculturation on breastfeeding initiation and early complimentary feeding. Breastfeeding initiation in the non-Irish cohort reduced from 89.4 of mothers who had arrived within the last year to five years ago to 67.5% for those who had arrived 11 to >20 years ago (*p* < 0.001). The percentage of non-Irish mothers introducing complimentary foods early increased from 6.6% for those who had arrived within the last year to five years ago to 16.0% for those who arrived 11 to >20 years ago (*p* < 0.001).

**Table 2:** Characteristics of Irish and non-Irish primary caregivers and households in the <17 weeks and ≥17 weeks complimentary feeding categories and binary logistic regression of the significant factors associated with the introduction of complimentary feeding.

Characteristics	Complimentary Feeding Introduction *											
	Total		<17 Weeks		≥17 Weeks		Unadjusted ^†^		Adjusted ^‡^			
	*n*	% ^γ^	*n*	% ^η^	*n*	% ^η^	OR	*p* ^§^	OR	95% CI		*p* ^δ^
*Primary caregiver age (years)*												
≥35	3467	31.8	426	12.3	3041	87.7	0.467		0.606	0.494	0.744	<0.001
25–34	6119	56.1	823	13.4	5296	86.6	0.519		0.664	0.555	0.794	<0.001
≤24	1321	12.1	304	23	1017	77	1.0 ^$^	<0.001	1.0 ^$^			<0.001
*Maternal education*												
Third level education	5330	48.9	557	10.5	4773	89.5	0.534		0.82	0.602	1.117	0.208
Secondary level education	5189	47.6	926	17.8	4263	82.2	0.994		1.031	0.773	1.374	0.835
No formal or primary education	378	3.5	68	18	310	82	1.0^$^	<0.001	1.0 ^$^			0.004
*Household class*												
Professional, managerial and technical workers	5228	47.9	578	11.1	4650	88.9	1.0 ^$^		1.0 ^$^			0.001
Non-manual	1983	18.2	332	16.7	1651	83.3	1.62		1.179	1.001	1.387	0.048
Skilled and semi-skilled manual	2428	22.3	377	15.5	2051	84.5	1.478		1.111	0.941	1.311	0.214
Unskilled and all other gainfully occupied and unknown	283	2.6	66	23.3	217	76.7	2.43		1.85	1.346	2.544	<0.001
Never worked at all	985	9	200	20.3	785	79.7	2.051	<0.001	0.972	0.752	1.258	0.83
*Household type*												
One parent one child under 18 years	799	7.3	177	22.2	622	77.8	1.042		0.925	0.72	1.189	0.545
One parent two or more children under 18 years	831	7.6	178	21.4	653	78.6	1.0^$^		1.0 ^$^			<0.001
Two parents one child under 18 years	3536	32.4	385	10.9	3151	89.1	0.447		0.614	0.479	0.788	<0.001
Two parents two or more children under 18 years	5740	52.6	813	14.2	4927	85.8	0.604	<0.001	0.827	0.659	1.038	0.101
*BMI primary carer*												
Underweight (less than 18.5 kg/m^2^)	298	2.7	43	14.4	255	85.6	1.132		0.903	0.639	1.274	0.569
Normal weight (18.5–24.9 kg/m^2^)	5519	50.6	714	12.9	4805	87.1	1.0 ^$^		1.0 ^$^			0.001
Overweight (25–29.9 kg/m^2^)	3264	29.9	456	14	2808	86	1.093		1.074	0.942	1.224	0.284
Obese (≥30 kg/m^2^)	1825	16.7	340	18.6	1485	81.4	1.542	<0.001	1.336	1.151	1.551	<0.001
*Primary carer ethnicity* *&* *length of stay in the ROI*												
Irish	9188	84.2	1424	15.5	7764	84.5	1.0 ^$^		1.0 ^$^			0.001
Non-Irish arrived within the last year-5 years ago	964	8.8	62	6.4	902	93.6	0.374		0.493	0.371	0.656	<0.001
Non-Irish arrived 6–10 years ago	570	5.2	44	7.7	526	92.3	0.456		0.566	0.408	0.785	0.001
Non-Irish arrived 11–>20 years ago	184	1.7	23	12.5	161	87.5	0.784	<0.001	0.969	0.613	1.532	0.893
*Exclusive breastfeeding duration*												
0–30 days	1579	14.5	241	15.3	1338	84.7	0.818		0.954	0.776	1.172	0.652
>30–60 days	770	7.1	101	13.1	669	86.9	0.687		0.864	0.665	1.122	0.272
>60–90 days	549	5	69	12.6	480	87.4	0.652		0.813	0.604	1.094	0.172
>90 days	1782	16.3	17	1	1765	99	0.044		0.061	0.037	0.101	<0.001
No exclusive breastfeeding	6227	57.1	1125	18.1	5102	81.9	1.0 ^$^	<0.001	1.0 ^$^			<0.001

* Bivariate analysis using *χ*^2^ statistical tests to compare the differences between primary caregivers, infants and households in the <17 weeks and ≥17 weeks groups. ^†^ Values are OR that were obtained from individual bivariate analysis of independent variables when compared to the dependent complimentary feeding variable <17 weeks and ≥17 weeks groups. ^γ^ Total percentage. ^η^ Percentage within each independent variable category who introduced complimentary feeding in the <17 weeks and ≥17 weeks groups. ^§^*P*-value resulting from unadjusted regression analysis of the independent variable with the complimentary feeding dependent variable <17 weeks and ≥17 weeks. ^‡^ Values are OR that were obtained from the final binary logistic regression model. **^δ^** *P*-values obtained from the adjusted binary logistic regression model. The model was adjusted for maternal age, education, BMI, SES, parity, mode of delivery, breastfeeding initiation, duration of exclusive breastfeeding, and infant's health status. 1.0 ^$^ Denotes the reference group.

## 4. Discussion

Breastfeeding is the most beneficial and nutritionally complete feeding method during infancy [30]. However, breastfeeding initiation rates in the ROI were the lowest compared to 14 European countries in 2010 [21]. Despite modest increases in breastfeeding rates, as shown in the Perinatal Statistics Report in 2012, these rates are still far from national targets and international averages [21,31,32,33,34].

In Table 1, it can be observed how a lower percentage of Irish mothers (49.5%) initiated breastfeeding compared to their non-Irish counterparts (88.1%). These findings correlate with previous studies in the ROI, which found similar percentages of breastfeeding initiation in the Irish and non-Irish mothers [23,26,27]. The percentages between the two cohorts are nearer to each other when mothers are asked about exclusive breastfeeding. However, the mean duration of any breastfeeding, as well as exclusive breastfeeding is lower for the Irish cohort. Both cohorts are far from complying with guidelines recommending six months of exclusive breastfeeding. However, a stronger predisposition towards breastfeeding, possibly due to cultural differences, is observed in the non-Irish group.

Figure 1 also shows that breastfeeding initiation was higher in all other ethnic groups when compared to the Irish cohort. The fact that any other white background has 86.5% breastfeeding initiation concurs with the study findings from 2010 in which Ireland had the lowest breastfeeding rates when compared to 14 European countries.

Differences in breastfeeding rates by ethnic background have been pointed out by other studies internationally [35,36,37,38,39]. Acculturation plays a role in infant feeding practices; as shown in Figure 2, the amount of non-national mothers initiating breastfeeding decreased the longer they had been living in the ROI (*p* < 0.001). Moreover, the percentage of these mothers introducing complimentary foods early also increased with a longer stay in the country (*p* < 0.001). This finding suggests the close relationship between the early milk feeding method chosen and the introduction of complimentary foods. Further exploration of the reasons behind these changes in infant feeding choices by non-Irish mothers is needed. Factors, such as societal pressures, language barriers and the perception of behaviours in the adopted culture as being modern, could potentially play a role in the acculturation mechanisms.

The relationship between acculturation and milk feeding choices has been reported by different studies in the United States (US) and Australia [40,41,42,43,44]. A study published in 2010 found that a group of Chinese mothers living in Ireland had a less positive attitude and more misconceptions about breastfeeding than a group of Chinese mothers living in Perth, Australia, suggesting a possible role of ‘acculturation’ and the mothers adapting themselves to the formula feeding culture of Ireland [45]. On the other hand, a 2013 study from Australia pointed out that Chinese mothers living in Perth had higher breastfeeding initiation rates and a longer duration of breastfeeding than Chinese mothers in Chengdu. Reported breastfeeding initiation rates in Australia are much higher than in the ROI [31,46]. These findings suggest that the culture of the adopted country may be an important influence on infant feeding practices among migrants.

Lack of breastfeeding and the use of formula feeding have been related to early complimentary feeding by many studies [14,15,17,18]. Formula feeding has been associated with impairment of appetite self-regulatory mechanisms, leading to infants demanding the introduction of solids earlier, with no subsequent reduction in milk intake during the complimentary feeding period. This interference with self-regulating mechanisms early in life could have long-term health consequences, increasing the risk of being overweight and obesity later in life [7,8,13,47].

Several studies have linked early complimentary feeding to a higher risk of being overweight and obesity during childhood and later in life [6,7,9]. An analysis of the same cohort at three years of age found that those children who were introduced to complimentary feeding later had a lower prevalence of being overweight or obesity [48]. Previous studies on complimentary feeding in the ROI have shown poor compliance with current guidelines, with more than 70% of infants being introduced to complimentary foods <17 weeks [15]. However, these studies did not explore ethnic variations in complimentary feeding.

An important finding in this study is observed in Figure 2, which shows that a higher percentage of Irish mothers (15.5%) introduced complimentary foods early when compared to the other ethnic groups. It has to be noted that the prevalence of infants introduced early to complimentary foods in this study is probably an underestimation, because mothers were asked for the child’s age at which point solid foods had been regularly given. The group with the lowest percentage of mothers introducing complimentary foods early were those of Chinese or any other Asian background (4.8%). Interestingly, this was one of the ethnic groups with one of the highest breastfeeding rates, which suggests a close relationship between early milk feeding and complimentary feeding.

The predictors of early complimentary feeding were studied for the whole sample. An important finding is that belonging to a different ethnic background than Irish had a protective role against early complimentary feeding, which was reduced with a longer length of stay in the ROI (Table 2). Figure 3 shows how the acculturation of non-Irish mothers resulted in a decrease in the breastfeeding rate, which correlates with an increase in the percentage of mothers introducing early complimentary foods. This finding highlights again the role played by acculturation and the adoption of formula milk in the timing of complimentary feeding introduction.

The inclusion of the duration of exclusive breastfeeding in the adjusted model resulted in a loss of the significance of breastfeeding initiation with little change in the rest of the significant predictors. This result suggests the importance of exclusive breastfeeding and its potential role in the timing of solids introduction and, ultimately, in the development of being overweight and obesity.

There is inconsistency in the results of studies on early complimentary feeding and the risk of developing being overweight and obesity. Moreover, a longer duration of breastfeeding is associated with the later introduction of complimentary foods. In the present study, a longer duration of exclusive breastfeeding resulted in a decrease in the probability of early complimentary feeding. Therefore, complimentary feeding could potentially be a confounder in the relationship between breastfeeding and being overweight or obesity [49,50,51,52].

Another interesting finding was the fact that maternal BMI was a predictor of early complimentary feeding. The relationship between being overweight, obesity and breastfeeding duration has been well studied, suggesting that overweight and obese women are at higher risk of early cessation of breastfeeding, due to biological and mechanical factors. [53,54,55,56].

## 5. Strengths and Limitations

GUI is a large and nationally representative sample. The results of the study can be applied at a population level, due to the application of the sampling weights. Parental BMI was measured by trained professionals using validated techniques.

However, there are several limitations to the present study. It would have been desirable to collect information on the first introduction of solids into the infant’s diet to allow comparability with other studies on complimentary feeding. The results must also be interpreted with caution, as the information was collected retrospectively, when the infant was nine months of age, increasing the possibility of recall bias.

Maternal BMI was measured at the time of interview, which took place when the infant was nine months old. Therefore, we assume that those mothers who were overweight or obese at that point in time belonged to the same BMI category pre-pregnancy.

## 6. Conclusions

The results from this study suggest that, after adjusting for other maternal characteristics, inappropriate infant feeding practices are more common among Irish mothers when compared to non-Irish mothers residing in the ROI. Acculturation plays an important role in infant feeding practices among non-Irish mothers. Therefore, cultural differences are an important factor in shaping patterns of infant feeding in the ROI.

There is a strong association between breastfeeding and the early introduction of complimentary feeding. The ROI continues to have one of the lowest breastfeeding rates in the world. Existing policies to increase breastfeeding rates have been largely ineffective and with recent increases in the breastfeeding rate explained by an increase in the proportion of non-Irish mothers residing in the ROI and increasing maternal education and age, characteristics that are associated with a higher propensity to breastfeed in Ireland. The immediate revision of current support, education and policies on infant feeding practices would appear desirable to achieve the implementation of desirable infant feeding practices in line with WHO and ESPGHAN recommendations.
